# Peritumoral ductular reaction can be a prognostic factor for intrahepatic cholangiocarcinoma

**DOI:** 10.1186/s12876-020-01471-0

**Published:** 2020-10-02

**Authors:** Zhenyang Shen, Jingbo Xiao, Junjun Wang, Lungen Lu, Xinjian Wan, Xiaobo Cai

**Affiliations:** 1 Department of Gastroenterology, Shanghai General Hospital, Shanghai Jiao Tong University School of Medicine, Shanghai, China; 2 Department of Gastroenterology, Shanghai Sixth People’s Hospital, Shanghai Jiao Tong University School of Medicine, Shanghai, China

**Keywords:** Peritumoral, Ductular reaction, Prognosis, Intrahepatic cholangiocarcinoma

## Abstract

**Background:**

Peritumoral ductular reaction (DR) was reported to be related to the prognosis of combined hepatocellular-cholangiocarcinoma and hepatocellular carcinoma. Non-mucin-producing intrahepatic cholangiocarcinoma (ICC) which may be derived from small bile duct cells or liver progenitor cells (LPCs) was known to us. However, whether peritumoral DR is also related to non-mucin-producing ICCs remains to be investigated.

**Methods:**

Forty-seven patients with non-mucin-producing ICC were eventually included in the study and clinicopathological variables were collected. Immunohistochemical analysis and immunofluorescence staining for cytokeratin 19, proliferating cell nuclear antigen, and α-smooth muscle actin were performed in tumor and peritumor liver tissues.

**Results:**

A significant correlation existed between peritumoral DR and local inflammation and fibrosis. (*r* = 0.357, 95% CI, 0.037–0.557; *P* = 0.008 and *r* = 0.742, 95% CI, 0.580–0.849; *P <* 0.001, respectively). Patients with obvious peritumoral DR had high recurrence rate (81.8% vs 56.0%, *P* = 0.058) and poor overall and disease-free survival time (*P* = 0.01 and *P* = 0.03, respectively) comparing with mild peritumoral DR. Compared with the mild peritumoral DR group, the proliferation activity of LPCs/ cholangiocytes was higher in obvious peritumoral DR, which, however, was not statistically significant. (0.43 ± 0.29 vs 0.28 ± 0.31, *P* = 0.172). Furthermore, the correlation analysis showed that the DR grade was positively related to the portal/septalα-SMA level (r = 0.359, *P* = 0.001).

**Conclusions:**

Peritumoral DR was associated with local inflammation and fibrosis. Patients with non-mucin-producing ICC having obvious peritumoral DR had a poor prognosis. Peritumoral DR could be a prognostic factor for ICC. However, the mechanism should be further investigated.

## Background

Cholangiocarcinoma (CCA) is highly malignant with a 5-year survival rate of 0 to 10% [1]. CCA can be divided mainly into extrahepatic cholangiocarcinoma and intrahepatic cholangiocarcinoma (ICC) based on the anatomic features [2]. ICC accounts for approximate 10% of primary liver cancer, and the incidence continues to increase in recent years [3]. ICC can also be divided into two categories: mucin producing and non-mucin producing, which have different pathological characteristics and origins. Although ICC is thought to develop from the intrahepatic bile duct, at least some ICCs, especially non-mucin-producing ICCs, may be derived from small bile duct cells or liver progenitor cells (LPCs) [4, 5]. In combined hepatocellular-cholangiocarcinoma may originate from bipotential LPC due to their dual characteristics of cholangiocytes and hepatocytes, background LPC was reported to be a prognostic factor after resection due to the origin of tumor cells [6]. Ductular reaction (DR) consisting of LPCs or small cholangiocytes represents hepatic or biliary regeneration in the liver. Peritumoral DR consisting of LPCs or small cholangiocytes is related to the prognosis of combined hepatocellular-cholangiocarcinoma and hepatocellular carcinoma [6, 7]. However, whether similar finding can also be observed in non-mucin-producing ICC remains to be investigated.

In the present study, patients with non-mucin-producing ICC were included, and the extent of peritumoral DR was evaluated to explore whether a relationship existed between DR and prognosis of ICC as well as its potential mechanism.

## Methods

### Patients and specimens

From January 2004 to December 2016, 47 patients with ICC, who underwent curative surgery in Shanghai General Hospital, Shanghai Jiao Tong University School of Medicine were included in the study. The written informed consent was obtained from each patient under a protocol approved by the ethics committee of Shanghai General Hospital. The tumor stage was determined according to the 2009 UICC TNM classification system [8]. The tumor and peritumor (< 2 cm away from the tumor) tissues from each patient and the available nontumor tissues (> 2 cm away from the tumor) from 30 patients were paraffin embedded.

### Histological and immunohistochemical analysis

**Fig. 1:**
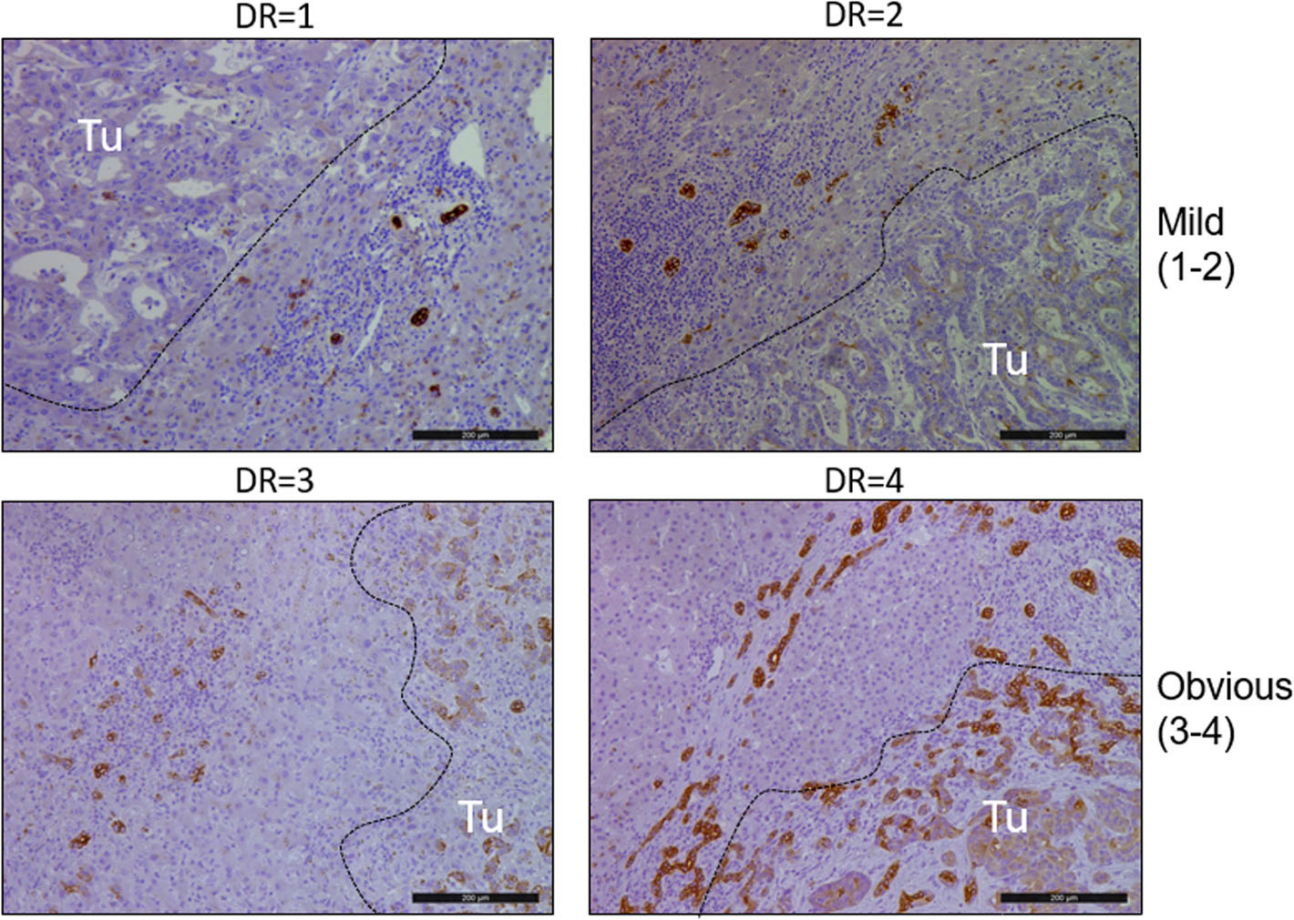
Representative figures showing different grades of peritumoral DR indicated by immunohistochemical staining for CK19 in ICC (scale bar = 200 μm)

Liver tissues were fixed in 4% formaldehyde and embedded in paraffin. Sections (4 μm thick) were stained with hematoxylin and eosin stain. According to the Scheuer scoring system, the inflammation and fibrosis of the peritumor tissues were independently scored by two experienced pathologists. If the scores of two pathologists were inconsistent, we had asked the third experienced pathologist to score and made the decision [9]. For immunohistochemical analysis, having been washed with phosphate-buffered saline, the sections were transferred into 10 mM sodium citrate buffer (pH 6.0), and antigen unmasking was performed in a microwave. After cooling down, the sections were incubated with peroxidase blocking reagent (Dako, Hamburg, Germany) for 1 h and then stained overnight at 4 °C with the following primary antibodies: anti-cytokeratin 19 (CK19) (Dako), 1:200; anti-proliferating cell nuclear antigen (PCNA) (Santa Cruz Biotechnology, USA), 1:200. The sections were developed with diaminobenzidine for 5 mins. The DR grade was evaluated according to the following standards: 0, no or minimal DR around a few portal tracts and septa; 1, focal DR around most portal tracts/septa; 2, continuous DR around < 30% of portal tracts/septa; 3, continuous DR around 30–50% of portal tracts/septa; and 4, continuous DR around more than 50% of portal tracts/septa (Fig. 1). For quantitative analysis, mean values of immunoreactive cells by counting the three fields including the portal or septal area at 200 × magnification were randomly obtained. The proliferation index (PI) was calculated as the ratio between the number of PCNA^+^ cells and the total number of reactive ductular cells or tumor cells. The high PI means that the ratio of the number of PCNA^+^ cells in number of reactive ductular cells or tumor cells is > 50%.

### Double-fluorescence immunostaining

Double-fluorescence immunostaining of formalin-fixed and paraffin-embedded tissue was performed using a sequential fluorescence method as previously described [10]. Alexa488 or Alexa647-conjugated goat antirabbit antibody (Invitrogen) was used as the secondary antibody. Nuclei were stained with 4′,6-diamidino-2-phenylindole (DAPI). Immunofluorescence was observed using Olympus IX-71 inverted microscope.

### Statistical analysis

Data were analyzed using SPSS version 23.0 for Windows (SPSS Inc., Chicago, IL, USA) and presented as means and standard deviations (±SD). Student *t* tests were used to compare the continuous quantitative data. A two-tailed Wilcoxon signed-rank test was used to compare ranked variables. The correlation between the degree of DR and clinicopathological variables was determined by Spearman or Pearson correlation as appropriate. The overall survival and disease-free survival were analyzed via the Kaplan-Meier method and compared using the log rank test. Multivariate Cox proportional hazard regression analyses were performed to identify risk factors for overall survival and disease-free survival. *P* value < 0.05 was considered statistically significant.

## Results

### Clinicopathological and follow-up data

Forty-seven patients (30 men and 17 women) were eventually included in the present study, with a mean age of 58.4 ± 11.0 years. Nine patients had lymph node invasion, and five patients had distant metastasis at the time of diagnosis. After a follow-up period of 25.7 ± 19.1 months, 32 patients (68.1%) developed intrahepatic recurrence after surgery (Table 1).

**Table 1:** The comparison of clinicopathological parameters between mild and obvious peritumoral DR patients

	Mild DR (*n* = 25)	Obvious DR (*n* = 22)	*P* value
Age	59.3 ± 9.4	57.4 ± 12.8	0.489
Gender			0.560
Male	15 (60.0%)	15 (68.1%)	
Female	10 (40.0%)	7 (31.9%)	
TNM stages			0.724
I-II	8 (32.0%)	6 (27.3%)	
III-IV	17 (68.0%)	16 (72.7%)	
T			0.491
1–2	10 (40.0%)	11 (50.0%)	
3–4	15 (60.0%)	11 (50.0%)	
N			0.559
0	21 (84.0%)	17 (77.3%)	
1	4 (16.0%)	5 (22.7%)	
M			0.532
0	23 (92.0%)	19 (86.4%)	
1	2 (8.0%)	3 (13.6%)	
Differentiation			0.510
1–2	16 (64.0%)	12 (54.5%)	
3–4	9 (36.0%)	10 (45.5%)	
Recurrence			0.057
No	11 (44.0%)	4 (18.2%)	
Yes	14 (56.0%)	18 (81.8%)	

### Peritumoral DR was related to local inflammation and fibrosis

**Fig. 2:**
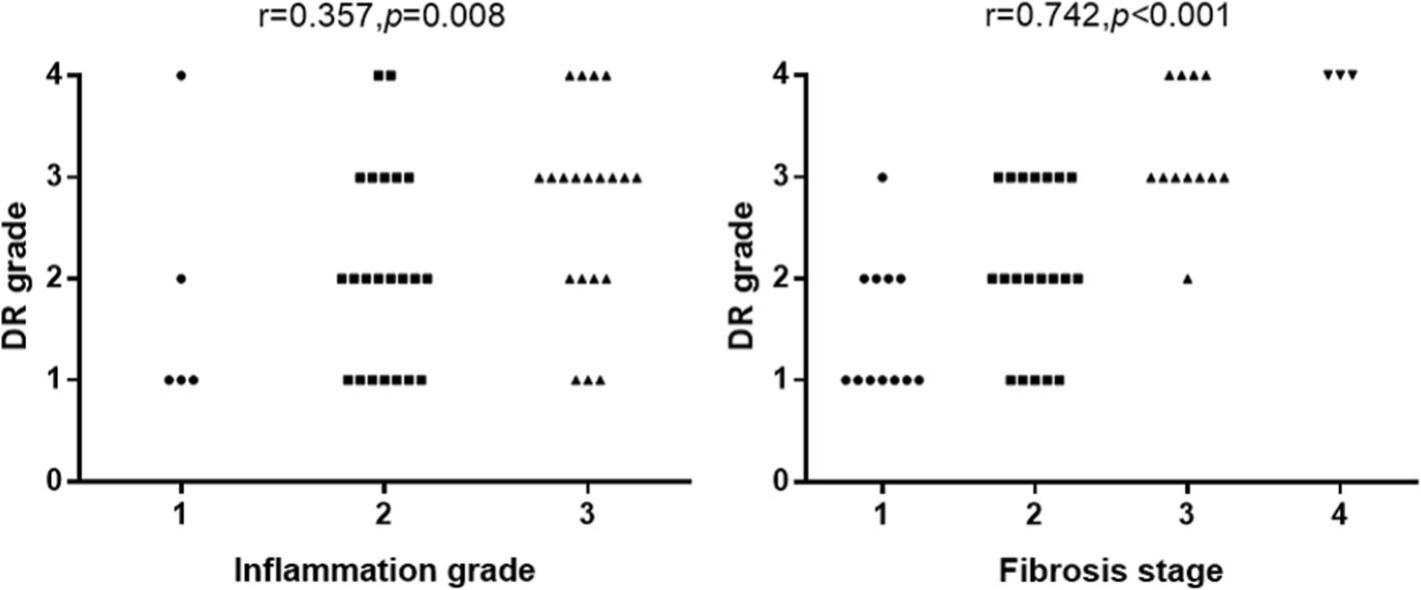
Grade of DR was closely correlated with local inflammation and fibrosis in peritumoral liver tissues

**Fig. 3:**
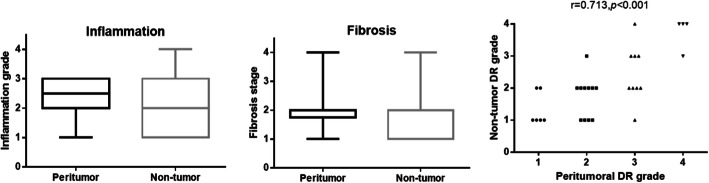
Comparison of local inflammation, fibrosis, and DR grade between peritumoral and nontumor areas

The correlation analysis showed that peritumoral DR was significantly correlated with local inflammation and fibrosis (*r* = 0.357, 95% CI, 0.037–0.557; *P* = 0.008 and *r* = 0.742, 95% CI, 0.580–0.849; *P <* 0.001, respectively) (Fig. 2). Due to the different definition of background DR, the DR grade in peritumor (< 2 cm away from the tumor) and nontumor (> 2 cm away from the tumor) areas was also compared in 30 patients whose liver tissues from both sites were available. The results demonstrated a small difference in local inflammation and fibrosis between them, but with no statistical significance. The DR grade in nontumor area is positively correlated with that in peritumoral area (*r* = 0.713, 95% CI, 0.499–0.862; *P* < 0.001) (Fig. 3). These results indicated a similar extent of DR and local environment between peritumor and nontumor areas.

### Peritumoral DR was related to the prognosis of ICC

**Fig. 4:**
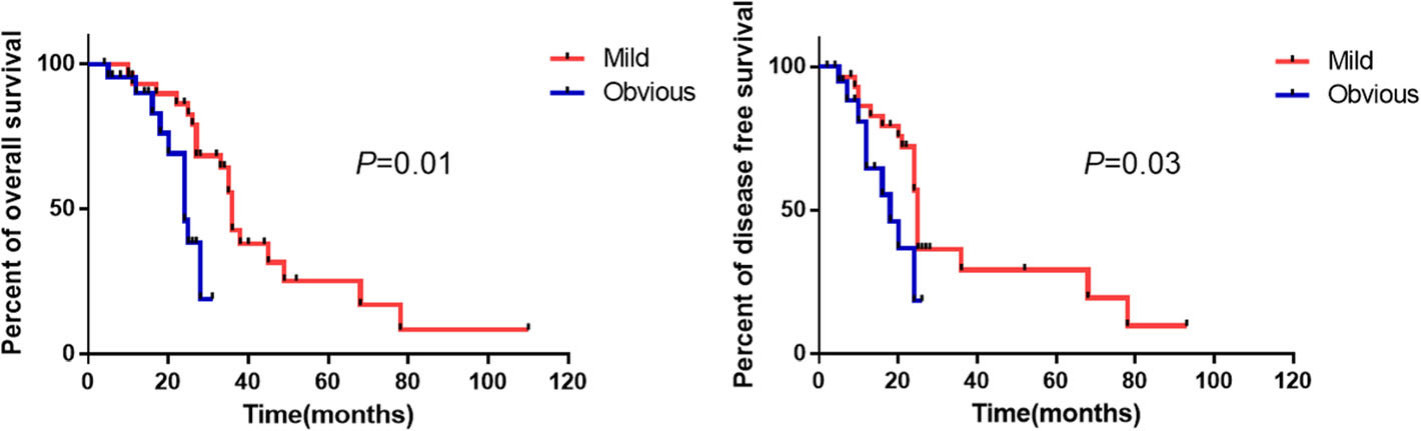
Kaplan–Meier analysis of the overall and disease-free survival of patients with ICC having different grades of DR

According to the grade of peritumoral DR, patients with ICC were divided into two groups: mild peritumoral DR (grades 1 and 2) (*n* = 25) and obvious DR (grades 3 and 4) (*n* = 22). Age, gender composition, TNM stages, and tumor differentiation were not significantly different between these two groups. However, the trend of tumor recurrence in the obvious DR group was much higher than that in the mild DR group but it was not statistically significant (81.8% vs 56.0%, *P* = 0.058) (Table 1). The survival analysis showed that patients with obvious peritumoral DR had poor overall and disease-free survival (*P* = 0.01and *P* = 0.03, respectively) (Fig. 4). In the multivariate analysis, obvious peritumoral DR was negatively associated with overall survival and disease-free survival (95% CI, 0.042–0.588; *P* = 0.016 and 95% CI, 0.072–0.647; *P* = 0.013, respectively) (Supplementary Table 1–2).

### Different proliferation of peritumoral ductular cells

**Fig. 5:**
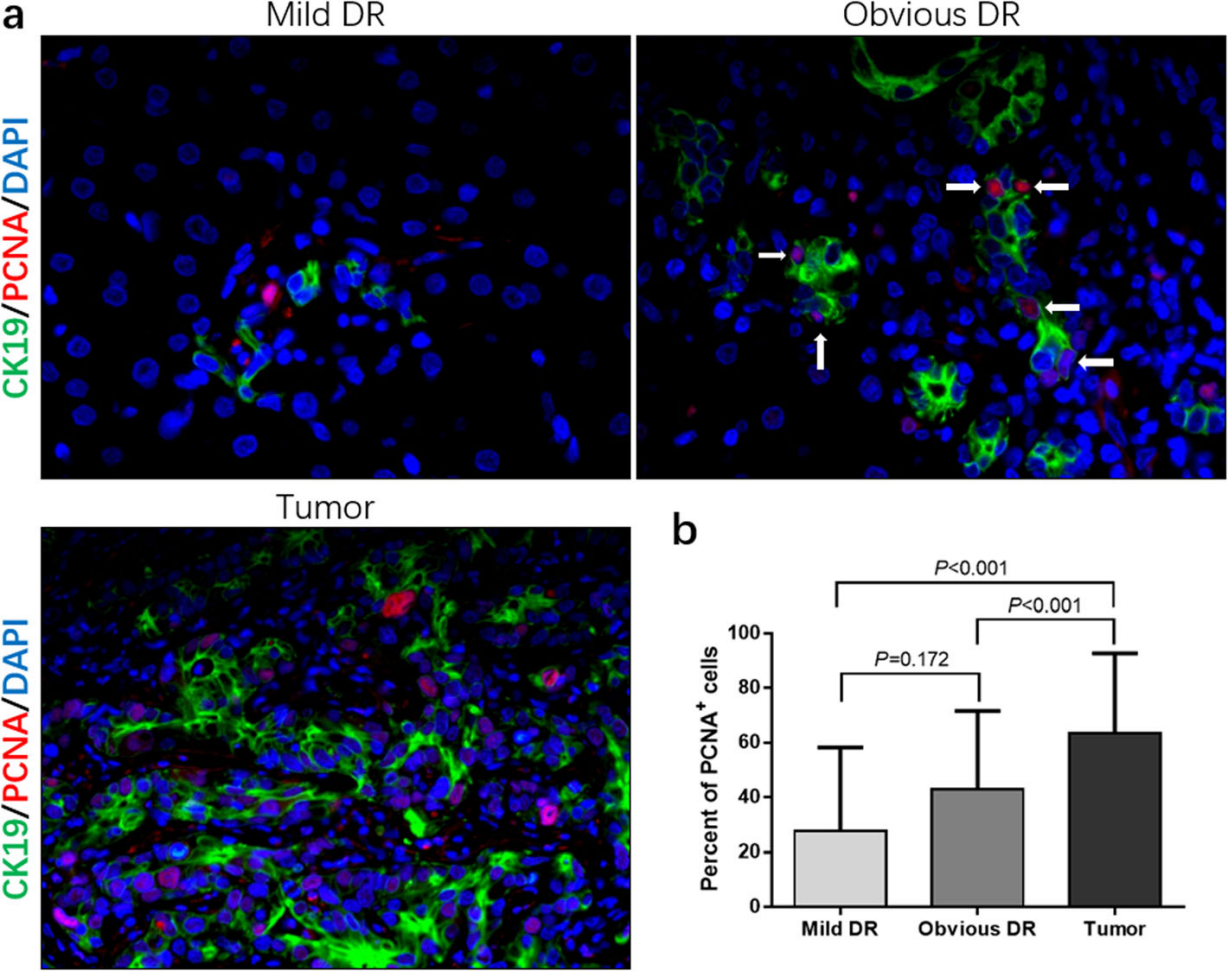
PI in the different peritumoral DR groups and tumor group. a CK19/PCNA positive expression in the mild and obvious peritumoral DR group and tumor group. b The obvious peritumoral DR group indicated a higher PI trend of ductular cells compared with the mild peritumoral DR group, and tumor group showed much higher PI compared with the other two groups. [PI = number of PCNA-positive (arrow)/total number of reactive ductular/tumor cells]

According to the previous description, PI was used to mark the extent of proliferation (Fig. 5a). The obvious peritumoral DR group showed a higher PI trend of ductular cells compared with the mild peritumoral DR group but failed to achieve statistical significance due to high variation (0.43 ± 0.29 vs 0.28 ± 0.31, *P* = 0.172). The percentage of high PI (> 50%) ductular cells was also higher in the obvious peritumoral DR group (44.44% vs 30.77%, *P* < 0.01). Undoubtedly, the tumor cells showed much higher PI compared with the other two groups (Fig. 5b).

### Different grade of peritumor DR was related to different microenvironments

**Fig. 6:**
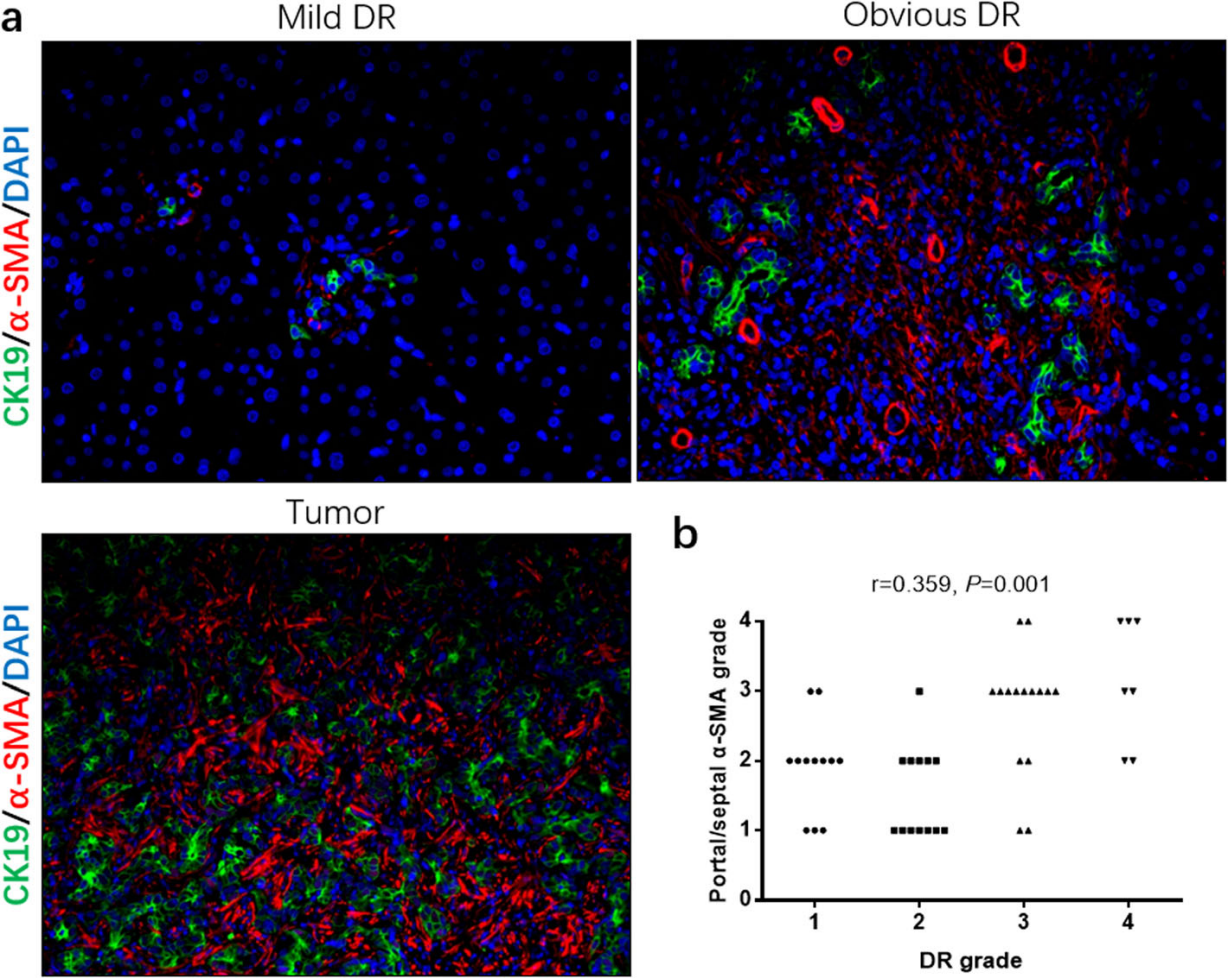
Microenvironment in the different peritumoral DR groups and tumor group. **a** The obvious DR group had more abundant ECM and α-SMA-positive vessels in peritumoral areas than in the mild DR group, and tumor group had similar microenvironment with the obvious DR group. **b** The correlation analysis illustrated that the DR grade was positively related to the portal/septal α-SMA level

ICC is a kind of tumor with abundant extracellular matrix (ECM), which plays an indispensable role in tumor progression. Double-fluorescence immunostaining showed the α-smooth muscle actin (α-SMA)-positive fibrosis background and CK19-positive ductular and tumor cells. The results demonstrated that there were more abundant ECM andα-SMA-positive vessels in peritumoral areas of obvious DR group than in mild DR group, which was similar to that in the tumor (Fig. 6a). The correlation analysis showed that the DR grade was positively related to the portal/septalα-SMA level (r = 0.359, *P* = 0.001) (Fig. 6b).

## Discussion

ICC can pathologically be divided into two categories: mucin producing and non-mucin producing. The former is derived from large intrahepatic bile ducts and has pathological features which are similar to those of extrahepatic cholangiocarcinoma, while the latter is considered to be derived from LPCs or small bile duct cells [4, 5]. ICC and hepatocellular carcinoma share some common risk factors, providing evidence that some ICCs might originate from bipotential LPC. A recent meta-analysis showed that cirrhosis and hepatitis B or hepatitis C virus (HCV) infection was a potential risk factor with the odds ratio value of 22.92, 5.1, and 4.8 respectively [11]. Another study showed that HCV infection and cirrhosis were the main risk factors for ICC [12]. On the contrary, liver fibrosis and inflammation were also main factors influencing the extent of DR in chronic liver diseases [13–15]. Therefore, it was speculated that peritumoral DR might be related to ICC due to common influencing factors and cell origins.

For combined hepatocellular-cholangiocarcinoma derived from LPC, the active peritumoral LPC was considered to be related to recurrence after resection [6]. Peritumoral DR was also correlated with the prognosis in hepatocellular carcinoma [7]. However, the relationship between peritumoral DR and prognosis of ICC is still not elucidated. Because non-mucin-producing ICC may be derived from small cholangiocytes or LPCs, which are the main source of DR, their relationship was studied. In the present study, the peritumoral DR was closely related to local liver inflammation and fibrosis, just like in chronic liver disease. Also, a similar extent of DR and liver inflammation or fibrosis was found in peritumor and nontumor areas (< 2 or > 2 cm from the tumor), indicating that such microenvironment was not just located in peritumoral areas.

According to the grade of peritumoral DR, patients with ICC were divided into two groups: mild DR and obvious DR groups. The clinical and pathological variables were compared between the two groups, and the results showed that the latter had a higher recurrence rate compared with the former. The survival analysis showed that patients with obvious peritumoral DR had significant poor overall and disease-free survival time. Hence, it could be concluded that peritumoral DR was related to non-mucin-producing ICC and could be a prognostic factor. Because the sample was not adequately powered to detect differences, the clinical outcomes may have been affected. Studies with larger sample sizes are warranted to confirm our results.

The mechanism underlying the relationship between peritumor DR and ICC is still not clear. A study demonstrating the correlation of peritumor DR with the prognosis of combined hepatocellular-cholangiocarcinoma speculated that peritumoral LPC probably provided the “field effect” and led to the development of tumor [6]. The present study also showed that peritumoral DR was related to ICC occurrence and poor prognosis. However, it is still hard to elucidate whether the activated peritumor LPCs/cholangiocytes could cause tumor occurrence. Immunostaining by PCNA showed that the proliferation activity of LPC was significantly enhanced in the obvious DR group than in the mild DR group. ICC is characterized by its abundant ECM, and the tumor-related macrophages and fibroblasts located in the ECM are related to poor prognosis. Immunostaining byα-SMA also demonstrated that significantly abundant ECM and vessels were accompanied by obvious DR, indicating that obvious peritumoral DR might share similar microenvironment with ICC. To sum up, peritumoral DR offered “field effect” which is likely to affect a wide area of the target tissue. In this study, we demonstrated that obvious peritumoral DR not only shared some characteristics with ICC but also provided insight into the recurrence of ICC in patients who have undergone curative surgery.

## Conclusions

The present study showed that patients with ICC having obvious peritumoral DR had a poor prognosis. Obvious peritumoral DR had a high proliferation activity of LPCs/cholangiocytes and abundant background ECM, which was similar to ICC. Although it is unclear whether activated peritumoral LPCs/cholangiocytes could lead to tumor occurrence or only be the result of fibrosis, which is also a risk factor of ICC, the results suggest that peritumoral DR could be a prognostic factor for ICC. However, studies with larger sample sizes are needed to confirm our results and the mechanism should be further investigated.

## Supplementary information

**Additional file 1: Supplementary Table 1.** Multivariate analysis of overall survival. **Supplementary Table 2.** Multivariate analysis of disease-free survival.

## Data Availability

The datasets generated or analyzed during the current study are available from the corresponding author on reasonable request.

## Abbreviations

DR: Peritumoral ductular reaction
ICC: Intrahepatic cholangiocarcinoma
LPC: Liver progenitor cells
CK19: Cytokeratin 19
PCNA: Proliferating cell nuclear antigen
α-SMA: Alpha-smooth muscle actin
CI: Confidence interval
CCA: Cholangiocarcinoma
UICC: Union Internationale Contre le Cancer
PI: Proliferation index
DAPI: 4′,6-diamidino-2-phenylindole
SD: Standard deviations
ECM: Extracellular matrix
HCV: Hepatitis C virus
HR: Hazard ratio
