# Supplementary material for: Peritumoral ductular reaction can be a prognostic factor for intrahepatic cholangiocarcinoma
Source: BMC Gastroenterol. 2020 Oct 2;20:322. doi: 10.1186/s12876-020-01471-0 (PMC7532600; doi:10.1186/s12876-020-01471-0)
Supplement: Supplementary file 1 — Additional file 1: Supplementary Table 1. Multivariate analysis of overall survival. Supplementary Table 2. Multivariate analysis of disease-free survival. [file 12876_2020_1471_MOESM1_ESM.docx]

|  | Univariate | | Multivariate | |
| --- | --- | --- | --- | --- |
| Variable | HR (95% CI) | *P* value | HR (95% CI) | *P* value |
| Age(year) |  |  |  |  |
| ＜60 | 1.344(0.488-3.703) | 0.567 | - | - |
| ≥60 | 1.000 |  |  |  |
| Gender |  |  |  |  |
| Male | 1.112(0.393-3.144) | 0.842 | - | - |
| Female | 1.000 |  |  |  |
| TNM stages |  |  |  |  |
| I-II | 0.817(0.339-2.151) | 0.651 | - | - |
| III-IV | 1.000 |  |  |  |
| T |  |  |  |  |
| 1-2 | 0.810(0.305-2.151) | 0.672 | - | - |
| 3-4 | 1.000 |  |  |  |
| N |  |  |  |  |
| 0 | 1.483(0.264-8.351) | 0.655 | - | - |
| 1 | 1.000 |  |  |  |
| M |  |  |  |  |
| 0 | 0.603(0.127-2.859) | 0.524 | - | - |
| 1 | 1.000 |  |  |  |
| Differentiation |  | 0.478 | - | - |
| 1-2 | 1.227(0.441-4.652) |  |  |  |
| 3-4 | 1.000 |  |  |  |
| Recurrence |  | 0.029 |  | 0.042 |
| No | 0.626(0.519-0.958) |  | 0.701(0.489-0.987) |  |
| Yes | 1.000 |  | 1.000 |  |
| DR |  | 0.010 |  | 0.016 |
| 1-2 | 0.143(0.033-0.625) |  | 0.168(0.042-0.588) |  |
| 3-4 | 1.000 |  | 1.000 |  |

Supplementary Table 1. Multivariate analysis of overall survival

|  | Univariate | | Multivariate | |
| --- | --- | --- | --- | --- |
| Variable | HR (95% CI) | *P* value | HR (95% CI) | *P* value |
| Age(year) |  |  |  |  |
| ＜60 | 1.680(0.595-4.743) | 0.367 | - | - |
| ≥60 | 1.000 |  |  |  |
| Gender |  |  |  |  |
| Male | 0.895(0.286-2.078) | 0.242 | - | - |
| Female | 1.000 |  |  |  |
| TNM stages |  |  |  |  |
| I-II | 0.438(0.174-1.099) | 0.179 | - | - |
| III-IV | 1.000 |  |  |  |
| T |  |  |  |  |
| 1-2 | 0.640(0.205-1.592) | 0.272 | - | - |
| 3-4 | 1.000 |  |  |  |
| N |  |  |  |  |
| 0 | 1.498(0.498-7.684) | 0.884 | - | - |
| 1 | 1.000 |  |  |  |
| M |  |  |  |  |
| 0 | 0.703(0.457-1.854) | 0.196 | - | - |
| 1 | 1.000 |  |  |  |
| Differentiation |  | 0.482 | - | - |
| 1-2 | 1.567(0.578-4.584) |  |  |  |
| 3-4 | 1.000 |  |  |  |
| DR |  | 0.006 |  | 0.013 |
| 1-2 | 0.193(0.060-0.628) |  | 0.212(0.072-0.647) |  |
| 3-4 | 1.000 |  | 1.000 |  |

Supplementary Table 2. Multivariate analysis of disease-free survival
